# Phytochemical and Pharmacological Role of Liquiritigenin and Isoliquiritigenin From Radix Glycyrrhizae in Human Health and Disease Models

**DOI:** 10.3389/fnagi.2018.00348

**Published:** 2018-11-01

**Authors:** Mahesh Ramalingam, Hyojung Kim, Yunjong Lee, Yun-Il Lee

**Affiliations:** ^1^Well Aging Research Center, Daegu Gyeongbuk Institute of Science and Technology, Daegu, South Korea; ^2^Division of Pharmacology, Department of Molecular Cell Biology, Samsung Biomedical Research Institute, Sungkyunkwan University School of Medicine, Suwon, South Korea; ^3^Companion Diagnostics and Medical Technology Research Group, Daegu Gyeongbuk Institute of Science and Technology, Daegu, South Korea

**Keywords:** isoliquiritigenin, liquiritigenin, memory, Parkinson’s disease, Radix Glycyrrhizae

## Abstract

The increasing lifespan in developed countries results in age-associated chronic diseases. Biological aging is a complex process associated with accumulated cellular damage by environmental or genetic factors with increasing age. Aging results in marked changes in brain structure and function. Age-related neurodegenerative diseases and disorders (NDDs) represent an ever-growing socioeconomic challenge and lead to an overall reduction in quality of life around the world. Alzheimer’s disease (AD) and Parkinson’s disease (PD) are most common degenerative neurological disorders of the central nervous system (CNS) in aging process. The low levels of acetylcholine and dopamine are major neuropathological feature of NDDs in addition to oxidative stress, intracellular calcium ion imbalance, mitochondrial dysfunction, ubiquitin-proteasome system impairment and endoplasmic reticulum stress. Current treatments minimally influence these diseases and are ineffective in curing the multifunctional pathological mechanisms. Synthetic neuroprotective agents sometimes have negative reactions as an adverse effect in humans. Recently, numerous ethnobotanical studies have reported that herbal medicines for the treatment or prevention of NDDs are significantly better than synthetic drug treatment. Medicinal herbs have traditionally been used around the world for centuries. Radix Glycyrrhizae (RG) is the dried roots and rhizomes of *Glycyrrhiza uralensis* or *G. glabra* or *G. inflata* from the Leguminosae/Fabaceae family. It has been used for centuries in traditional medicine as a life enhancer, for the treatment of coughs and influenza, and for detoxification. Diverse chemical constituents from RG have reported including flavanones, chalcones, triterpenoid saponins, coumarines, and other glycosides. Among them, flavanone liquiritigenin (LG) and its precursor and isomer chalcone isoliquiritigenin (ILG) are the main bioactive constituents of RG. In the present review, we summarize evidence in the literature on the structure and phytochemical properties and pharmacological applications of LG and ILG in age-related diseases to establish new therapeutics to improve human health and lifespan.

## Introduction

The average life expectancy at birth in developed countries was about 47 years at the beginning of the 20th century and increased to 77.8 years in the beginning of the 21st century (Oeppen and Vaupel, 2002). Senescence or biological aging is neither irreversible nor inevitable fate of all organisms and significantly delayed (da Costa et al., 2016). The rate of dying prematurely has decreased, whereas the rate of death from aging-associated chronic diseases such as diabetes mellitus, cancer, and heart, kidney, and neurological diseases has increased (Fontana, 2009). The aging process in biological systems is complex (Gottfries, 1990) and is proving to be a major risk factor for all of the common chronic and lethal conditions. Aging results in marked changes in brain structure and function (Cole and Franke, 2017). Age-related neurodegenerative disorders (NDDs) represent an ever-growing socioeconomic challenge and lead to an overall reduction in quality of life around the world (Tarailo-Graovac et al., 2016).

Alzheimer’s disease (AD) and Parkinson’s disease (PD) are most common age-related neurological disorders of the central nervous system (CNS). AD is characterized by insoluble extracellular β-amyloid peptide (Aβ) forming senile plaques deposition, intraneuronal tau accumulation and hyperphosphorylated microtubule-associated fibrillary tangles. It may contribute to progressive neuronal degeneration with memory loss and cognitive impairment in brain during normal aging (Dickson et al., 1988). PD is characterized by loss of dopamine(DA)-rgic neurons and the presence of intracytoplasmic aggregated α-synuclein Lewy bodies in the substantia nigra pars compacta of the brain eventually leads to bradykinesia, rigidity, slowing of movement and postural instability (Gibb and Lees, 1988). In addition, low levels of brain neurotransmitter acetylcholine (ACh) (Houghton and Howes, 2005), oxidative stress, intracellular calcium ion imbalance, mitochondrial dysfunction, ubiquitin-proteasome system impairment and endoplasmic reticulum stress are involved in the pathogenesis of NDDs (Ramalingam and Kim, 2016b). Aging enables the development of chronic diseases, and anti-aging mechanisms not only increase lifespan but also preserve function resembling a more youthful state (Fontana, 2009). Therefore, there is a focus on developing novel multi-disease preventative and therapeutic approaches (Kennedy et al., 2014). At present, there are no cures for NDDs. The existing therapies are focused on increasing the amount of ACh and DA by inhibiting acetylcholinesterase and monoamine oxidase (MAO) inhibition, respectively or elevating the concentration of the precursor levodopa (L-DOPA) (Houghton and Howes, 2005).

The brain is the body’s most complex organ and not all drugs are sanctioned for the treatment of age-related NDDs. The currently available treatments have proven ineffective in curing the multi-functional pathological conditions of NDDs (Vijayakumar et al., 2016). Synthetically manufactured drugs have certain side effects, such as dry mouth, tiredness, sleepiness, sluggishness, drowsiness, anxiety, tension, nervousness, and trouble with balance in humans (Phani Kumar et al., 2015). Recently, increasing scientific researches on medicinal plants have widespread interest and their active ingredients may help to identify new multi-functional therapeutic agents. Numerous plant-based natural and their manufactured synthetic neuroprotective drugs have been permitted by the U.S. Food and Drug Administration (FDA) over the past 30 years (Kumar et al., 2012; Cragg and Newman, 2013). Plants have been used for many centuries in traditional medicines to treat NDDs (Abushouk et al., 2017). Dietary compounds present in daily foods such as fruits, seeds, vegetables, and beverages have been reported for neuroprotective by various signaling pathways against NDDs (Asthana et al., 2016). Although there are many medicinal plants documented in pharmacopeias, only a few have been extensively studied to understanding their potential effectiveness in the treatment of NDDs (Vijayakumar et al., 2016).

## *Glycyrrhiza uralensis* Fisch.

*Glycyrrhiza uralensis*, commonly known as licorice (or liquorice), is one of the most popular herbal medicines, used as antitussive, expectorant, and antipyretic in traditional prescriptions, foods, beverages, brewing, tobacco, and cosmetics for its effects of relieving cough, pharyngitis, bronchitis, and bronchial asthma (Cao et al., 2004; Kao et al., 2014). It is a perennial glandular herb, 30–100 cm high, with an erect stem with short whitish hairs and echinate glandular hairs; the lower part of the stem is woody. Leaves are alternate and imparipinnate, with 7–17 leaflets. Leaves are ovate-elliptical in shape and 2–5.5 cm long by 1–3 cm wide (WHO, 1999). The apex is obtuse-rounded and the base is rounded and both surfaces are covered with glandular hairs and short hairs. Stipules are lanceolate and inflorescence is an axillary cluster. Flowers are purplish and papilionaceous with villous calyx. The fruit is a flat oblong pod, 6–9 mm wide, that is sometimes falcate and is densely covered with brownish echinate glandular hairs. Each pod contains 2–8 seeds. The root is cylindrical, fibrous, flexible, 20–22 cm long and 15 mm in diameter, with or without cork, which is reddish, furrowed, and light yellow inside (WHO, 1999).

### Classification

Kingdom: Plantae (Plants)

Subkingdom: Tracheobionta (Vascular Plants)

Superdivision: Spermatophyta (Seed Plants)

Division: Magnoliophyta (Flowering Plants)

Class: Magnoliopsida (Dicotyledons)

Subclass: Rosidae

Order: Fabales

Family: Leguminosae/Fabaceae

Genus: *Glycyrrhiza*

Species: *G. uralensis* Fisch.

Binomial: *Glycyrrhiza uralensis* Fisch. Ex DC. (Theplantlist, 2013; Wikipedia, 2018)

### Synonyms

*Glycyrrhiza asperrima* var. *desertorum* Regel

*Glycyrrhiza asperrima* var. *uralensis* Regel

*Glycyrrhiza glandulifera* Ledeb. (Theplantlist, 2013)

### Vernacular Names

Chinese : Gan Cao (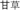)

Korean : Gam Cho (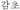)

Japanese : Uraru-kanzou (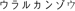)

## Radix Glycyrrhizae

Radix Glycyrrhizae (RG) is the dried roots and rhizomes of three Glycyrrhiza species—*G. uralensis* Fisch. ex DC., *G. glabra* L, and *G. inflata* Batalin—that is prescribed as licorice in Pharmacopeias (Liu et al., 2013; Wang et al., 2015). Among these species, *G. uralensis* is the most frequently used species for RG in Korea, China and Japan that constitutes 90% of total licorice production around the world (Zhang and Ye, 2009). Moreover, *G. uralensis* known as Far East Asian licorice and *G. glabra* as Western licorice (Davis and Morris, 1991), this review is limited to *G. uralensis*. The roots and rhizomes are cylindrical, fibrous, flexible, 20–100 cm long and 0.6–3.5 cm in diameter, with or without cork. Roots are externally reddish brown or grayish brown, longitudinally wrinkled, furrowed, and lenticellate, with sparse rootlet scars (WHO, 1999). The texture is compact and slightly fibrous, and the interior is yellowish white and starchy. The cambium ring is distinct and the rays radiate, some with clefts. Rhizomes are cylindrical with external bud scars, and pith is present in the center of the fracture (WHO, 1999).

RG is one of the most commonly used and oldest herbal medicines documented in the pharmacopeias (Liu et al., 2013). The earliest Chinese written literature *Shennong’s Classic of Materia Medica* describing the use of licorice dates from 2100 BC for its life-enhancing properties (Wang et al., 2015). Its clinical practice against cough, influenza, liver damage and for detoxification values has received considerable attention throughout the world (Ji et al., 2016). Recent researches revealed its antioxidant, anti-inflammatory, anti-viral, anti-diabetic, cytotoxic, skin-whitening and cholinergic activities (Mae et al., 2003; Isbrucker and Burdock, 2006; Nassiri and Hossein, 2008; Ahn et al., 2010). It is used as a natural sweetener and food additive in snacks, candies, cookies, seasoning sauce, soy sauce, and drinks (Kitagawa, 2002).

## Quality and Safety

Use of herbal medicines is an important tradition in rural communities for health and disease prevention (Fennell et al., 2004). However, there is always a risk of ineffectiveness, side effects, or misadministration of toxic plants. Identification, collection, processing, storage and contaminants in the natural plant products have also contributed to the deleterious effect in human body (Street et al., 2008). Quality, safety, and efficacy are the main concerns that must be evaluated in crude or fractionated extracts and their individual compounds and documented before they are prescribed for use as herbal medicines and botanical dietary supplements.

Licorice species have a unique profile of secondary metabolites and distinct biological activities (Li et al., 2016). Glycyrrhizin, present in all three species, is a sweet-tasting saponin that can cause hypertension, sodium salt and water retention, and reduced potassium ion levels (WHO, 1999; Farag et al., 2012), but the aqueous extract has less negative effects compared to glycyrrhizin pure compound (Bernardi et al., 1994; Cantelli-Forti et al., 1994). High doses of glycyrrhizic acid (400 mg/day) have risk of side effects, such as cardiac dysfunction, edema, and hypertension (Størmer et al., 1993).

The chemical ingredients may change during the extraction process of the herbs due to the solvents interaction or heating process is considered for their altered pharmacological effects (Wang et al., 2013). RG combined with Sargassum, Herba Cirsii Japonici, Euphorbia Kansui, and Flos Genkwa might cause cardiac toxicity (Huang et al., 2001). In contrast, RG reduces the toxicity of hydroxysafflor yellow A from Flos Carthami, strychnine and brucine from Semen Strychni, and aconite from Radix Aconiti Lateralis Praeparata (Wang et al., 2013). Therefore, systematic safety evaluation of prolonged use or overdose of compounds and potential drug–drug interactions is needed before the use of herbal medicine as a nutritional supplement and/or therapeutic drugs.

## Isoliquiritigenin and Liquiritigenin

The medicinal plants lies in their chemical constituents to produce physiological actions to have various health benefits in the human body (Sharma et al., 2018). As a result of genetic, ecological, and environmental differences, herbal medicines generally vary in their quality and chemical constituents (Bopana and Saxena, 2007). RG contains at least 400 different chemical constituents including triterpenoid saponins, flavanones, chalcones, coumarines, and their glycosides (Fujii et al., 2014; Kao et al., 2014). As there are many phytochemicals present in each plant, it is necessary to identify a single phytochemical that might be useful in the treatment of human health and diseases (Ramalingam and Kim, 2016a). However, little is known about the effective chemical constituents responsible for biological activities isolated from RG (Ji et al., 2016). More than 25% of the components in RG have been identified as active constituents through oral bioavailability, virtual screening and drug-likeness (Liu et al., 2013).

The chalcone isoliquiritigenin (ILG; 2′,4,4′-trihydroxychalcone) is biosynthetically and structurally interrelated to the flavanone liquiritigenin (LG; 4′,7-dihydroxyflavanone) (Simmler et al., 2013). These two polyphenols are the main bioactive constituents of RG (Table 1). ILG is the isomeric precursor of LG (Jayaprakasam et al., 2009). In flavonoid biosynthesis, chalcone isomerase (CHI) promotes enzymatic isomerization through which the ionized 2′-hydroxychalcones (e.g., ILG) is stereochemically converted to 2-S flavanone (e.g., LG). During this process, the ionized chalcone interacts with amino acid side chains in the CHI active site to produce flavanone by high enantioselectivity (Jez et al., 2000; Jez and Noel, 2002). Moreover, studies have reported that ILG and LG are interchangeable by pH and temperature. Chalcones undergo rapid cyclization into flavanones at neutral and lower acidic pH whereas flavones isomers undergo a reversible reaction to their chalcones at basic pH. This interconversion between chalcone and flavanone represents temperature dependent isomerization and racemization of these active compounds (Furlong and Nudelman, 1988; Miles and Main, 1989; Nudelman and Furlong, 1991; Cisak and Mielczarek, 1992; Yamín et al., 1997; Andújar et al., 2003). Interestingly, these chemical conditions match the cell culture-based assays also. LG and ILG were incubated in cell culture mediums such as RPMI 1640 or DMEM/F12 supplemented with 5 or 10% heat-inactivated fetal bovine serum maintained at pH around 7.4 in 5% CO_2_ at 37°C (with or without MCF-7 cells). ILG began isomerization and reached interconversion ratio of 10.4% ILG and 89.6% LG after 96 h. It suggests that LG and ILG have interconnected biological activities and the biological activity of ILG can be linked to its flavanone LG (Simmler et al., 2013).

**Table 1:** Chemical and interchangeable properties of ILG and LG.

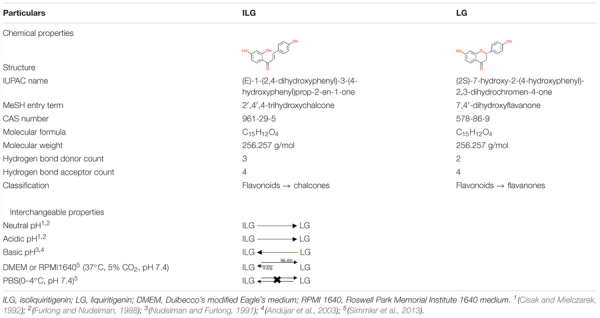

## Pharmacological Activities Against Ndds

Studies on bioactivities revealed that LG and ILG have some valuable pharmacological activities against NDDs.

### Antidepressant and Antianxiety Activities

Major depression is a severe life-threatening disorder caused by a complex pathophysiological process related to a person’s knowledge (Su et al., 2016; Tao et al., 2016). Lipopolysaccharide (LPS) has been used to cause acute depressive behavior as well as inflammation. Unpredictable chronic mild stress (UCMS) has been used as a model for chronic anxiety/depressive-like behavior. Male ICR mice were pretreated with LG at 7.5 and 15 mg/kg intragastrically for 7 days followed by subcutaneous injection of 0.5 mg/kg LPS. LG pretreatment significantly reduced the LPS-induced significant increase in immobility duration in forced swimming and tail suspension tests. Moreover, levels of the pro-inflammatory cytokines interleukin (IL)-6 (IL-6) and tumor necrosis factor-α (TNF-α) in serum and hippocampus were decreased in the LG group compared with the control LPS group. LG also decreased the expression of p-p65NF-κB, p-IκBα, brain-derived neurotrophic factor (BDNF), and p-TrkB (tropomyosin receptor kinase B). These results indicated that the antidepressant and antianxiety activities of LG might be associated with anti-inflammatory and BDNF/TrkB pathways (Su et al., 2016).

For the chronic type of anxiety, mice were exposed to UCMS for 6 weeks and intragastrically treated with LG (7.5 and 15 mg/kg) from the third week. LG-treated mice showed a reduced immobility time in forced swimming test (FST) and tail suspension test (TST) and a reduction in sucrose preference test (SPT). There were no significant changes in spontaneous locomotor activities. Levels of pro-inflammatory cytokines IL-6, IL-1β, and TNF-α in serum and hippocampus were downregulated by LG treatment. The elevated concentrations of glucocorticoids (GC) in plasma and serum malondialdehyde (MDA) were decreased, together with improved superoxide dismutase (SOD) and catalase (CAT) activity and glutathione (GSH) content. LG also upregulated the concentrations of 5-hydroxytryptamine (serotonin; 5-HT) and norepinephrine (NE) in hippocampus of mice. Different degrees of attenuation in BDNF and phosphorylated phosphoinositide 3-kinase (PI3K), protein kinase B (Akt), mammalian target of rapamycin (mTOR), and TrkB were observed. Collectively, these data showed that LG could alleviate depressive-like symptoms, possibly through regulation of the PI3K/Akt/mTOR–mediated BDNF/TrkB pathway in the hippocampus (Tao et al., 2016).

### Anti-psychostimulant Activities

Dopamine (DA) is a neurotransmitter in the CNS activated by drugs of abuse and psychostimulants such as cocaine (Jang et al., 2008; Jeon et al., 2008). It increases extracellular DA levels from dopaminergic neurons, resulting in spontaneous hyperlocomotion and other neurobehavioral changes. ILG inhibited cocaine-induced DA release in the nucleus accumbens of rat brain (Jeon et al., 2008). In addition, ILG caused alterations of a protooncogene protein c-Fos expression in cocaine-treated rat brain (White and Gall, 1987; Jang et al., 2008). Moreover, ILG treatment was associated with differences in gene expression patterns for 56 proteins including gamma enolase, glial fibrillary acidic protein (GFAP), and ubiquitin carboxyl-terminal hydrolase isozyme L1. These data suggest that ILG might be a potential agent against cocaine-induced neuronal cell injury and death (Jeon et al., 2008).

Activation of dopamine neurons by continued cocaine drug abuse that originate in the ventral tegmental area cause D1 and D2 DA receptor signaling dysfunction then leads to upregulation of c-Fos in the nucleus accumbens and prefrontal cortex (Bardo, 1998; Anderson and Pierce, 2005). Oral administration of ILG for 1 h prior to an injection of cocaine (20 mg/kg, i.p.) in male Sprague-Dawley rats suppressed the extracellular DA level in the nucleus accumbens in a dose-dependent manner and attenuated the expression of c-Fos. Gamma-aminobutyric acid (GABA)-β receptors attenuate the reinforcing effect of drugs. These effects of ILG were entirely stopped by a GABAβ receptor antagonist SCH 80911. These results suggest that ILG might be effective by modulating the GABAβ receptor to block the cocaine effects, and may be used in pharmacotherapy for drug addiction (Jang et al., 2008).

### Inhibitory Activities Against Monoamine Oxidases

The monoamine oxidases (MAOA and MAOB) catalyze the oxidative deamination of monoamines in the CNS and peripheral nervous system (PNS). MAO inhibitors are used in therapy for disorders of the CNS (Kanazawa, 1994). Licorice extracts (30 μg/ml) from different specimens were showed inhibitory effects on MAO without characterization of their active constituents (Hatano et al., 1991). LG (IC_50_: 32 μM) and ILG (IC_50_: 13.9 μM) inhibited the activity of MAOA in a dose-dependent manner, but non-competitively with the positive control clorgyline (IC_50_: 0.198 μM). The IC_50_ values for MAOB inhibition for LG, ILG, and positive control deprenyl were 104.6, 47.2, and 0.251 μM, respectively (Pan et al., 2000). These reports indicate that LG and ILG might be major MAO inhibitors in RG and useful for the treatment of anxiety and depression and the treatment and prevention of PD.

### Activities Against Parkinson’s Disease

PD is a progressive NDD described by the selective loss of dopaminergic (DArgic) neurons and formation of intracellular Lewy bodies such as α-synuclein (α-syn) within the substantia nigra. The α-syn monomers misfold into toxic oligomers of α-syn aggregates that subsequently increase the reactive oxygen species (ROS) level and induce apoptosis, resulting in dopaminergic neuronal injury in PD. *G. uralensis* is one of the main herbs in approximately 40% of traditional medical prescriptions described in pharmacopeias for the treatment of PD. ILG showed inhibitory effects against membrane toxicity in *in vitro* aggregated α-syn and disaggregated preformed mature fibrils in a transgenic *Caenorhabditis elegans* PD model (Liao et al., 2016).

Treatment of dopaminergic neuronal SN4741 cells with ILG at 0.1, 0.5, and 1 μM did not affect cell viability. Pretreatment with ILG (1 μM) attenuated 6-hydroxydopamine (6-OHDA)–induced cell death. ILG pretreatment significantly inhibited 6-OHDA–induced nuclear condensation, fragmentation and apoptosis, in accordance with inhibition of caspase-3 activation. ILG pretreatment completely attenuated generation of ROS and reactive nitrogen species (RNS), in addition to suppressing mitochondrial membrane potential (MMP) dissipation and cytochrome c in the cytosol. The protective effects of ILG appear to be mediated through attenuation of c-Jun N-terminal kinase (JNK), p38, and Akt by modulating the Bcl-2/Bax ratio, whereas the extracellular signal-regulated kinase 1/2 (ERK1/2) signaling pathway is not affected. ILG significantly enhanced production of BDNF, a neurotrophic factor, and reduced the downregulation of BDNF release that increases the survival and the morphological differentiation of dopaminergic neurons (Hwang and Chun, 2012). ILG (1 μM) pretreatment for 20 h followed for 24-h treatment with rotenone (3 μM) or sodium nitroprusside (SNP; 0.4 mM) in PC12 cells significantly improved MMP, adenosine triphosphate (ATP) level, and cell viability as well as cell proliferation (Denzer et al., 2016).

An E3 ubiquitin ligase, RNF146 recognizes its substrates through poly(ADP-ribosyl)ation (PARsylation) of proteins mediated by poly(ADP-ribose) polymerases (PARPs) (Kang et al., 2011; Zhou et al., 2011; Fatokun et al., 2014; DaRosa et al., 2015). RNF146 can disturb PARP1-regulated cellular processes and has neuroprotective activity against *N*-methyl-D-aspartate (NMDA)–induced excitotoxicity, DNA damage, and stroke. LG treatment at 10 μM increased 3-fold of RNF146 mRNA and protein expressions in SH-SY5Y cells and caused a nuclear translocation of endogenous estrogen receptor β (ERβ). Tamoxifen, an ER antagonist, blocked the LG-induced increase in RNF146 expression, indicating that ER activation was responsible for RNF146 induction. In addition, LG failed to induce RNF146 expression in an ERβ deletion model (Kim et al., 2017).

LG up to a concentration of 100 μM did not affect the viability of SH-SY5Y cells. In addition, LG completely eliminated the hydrogen peroxide (H_2_O_2_)–induced oxidative injury in SH-SY5Y cells. LG treatment (10 μM, 48 h) showed substantial protective effects on cell viability against PD-associated toxins 6-OHDA (70 μM, 24 h) and rotenone (20 μM, 24 h). In a model of H_2_O_2_ toxicity, primary mouse cortical neurons exposed to LG showed a 4-fold increase in RNF146 expression and reversed cellular ATP content and mitochondrial membrane potential. H_2_O_2_ induces robust PARP1 activation, which was largely abolished by LG treatment (Kim et al., 2017). AIMP2, a parkin substrate, enhances cell toxicity, and the levels of PAR-conjugated proteins were significantly reduced by LG-induced RNF146 expression. These effects collectively indicate that LG has various cytoprotective abilities against multiple PARP1-activating stimuli. shRNA-mediated silencing of RNF146 expression annihilated the LG-mediated cytoprotection against H_2_O_2_ injury in SH-SY5Y cells. Moreover, the shRNA-RNF146 expression knocked down the endogenous RNF146 expression by more than 80% and resulted in a sustained increase in PAR activity and depletion of ATP following H_2_O_2_ toxicity. Similarly, CRISPR-Cas9–mediated ERβ ablation eliminated the LG-induced neuroprotection against H_2_O_2_ toxicity. Taken together, these data indicate that LG-induced activations of ERβ and RNF146 inhibit PARP1 and confer neuroprotection (Kim et al., 2017).

**FIGURE 1:**
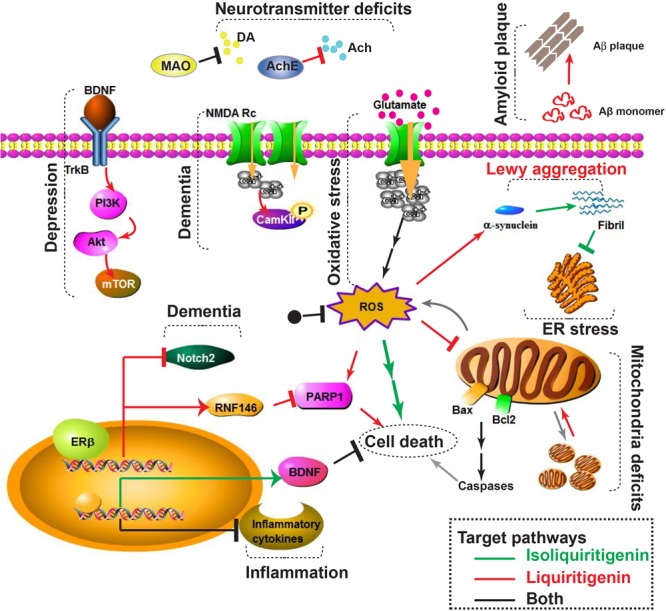
Diagrammatic representation of pathological phenotypes (depression, dementia, inflammation, oxidative stress, amyloid plaque, neurotransmitter deficits, Lewy aggregation, ER stress, mitochondrial deficits, and cell death) and underlying molecular pathways that could be involved in Alzheimer’s and Parkinson’s disease. Pathways regulated by isoliquiritigenin (ILG) and liquiritigenin (LG) were labeled as green and red lines, respectively. Pathways targeted by both ILG and LG were colored as black line. Arrows (↓) denote stimulation and bars (⊥) denote inhibition by ILG (green), LG (red), and both (black). PD related pathways include MAO (monoamine oxidase), and Lewy aggregation. AD related pathways include amyloid plaque formation and AChE (acetylcholine esterase). Other pathological processes are related to both AD and PD, thus could be therapeutic targets by ILG or LG. The scheme was generated by using Pathway Builder Tool.

As previously reported, LG can penetrate into the brain and exert neuroprotective activity (Yang E.J. et al., 2013). Intraperitoneal administration of LG to mice for 3 consecutive days resulted an increased levels of RNF146 mRNA and protein expressions in ventral midbrain, striatum, and cerebellum. In a 6-OHDA–induced PD-like model, LG pretreatment markedly enhanced tyrosine hydroxylase (TH)-positive DArgic neurons and almost completely inhibited PARP1 activity. Moreover, LG was shown to selectively activate ERβ, but not ERα, thus avoiding aberrant tumor growth *in vivo* (Mersereau et al., 2008) and improving the safety profile of long-term LG treatment for NDDs (Kim et al., 2017). Therefore, ILG and LG are potential candidates against PD in *in vitro* and *in vivo* models and their working mechanisms are diagrammatically represented in Figure 1.

### Memory Enhancing Activities

*N*-methyl-D-aspartic acid receptors (NMDARs) in the CNS have been allied to learning and memory (Nakazawa et al., 2004). Administration of LG at 20 mg/kg to ICR mice resulted in an increased level of spontaneous alternation behavior without an alternation in the number of arm entries. LG also significantly improved longer step-through latency time in a passive avoidance test. Regarding cognitive enhancement, LG influenced on hippocampal NMDAR subunits 1, 2A, and 2B expressions in ICR mice. For NMDAR downstream targets, LG significantly increased PSD-95 expression and phosphorylation of Ca^2+^/calmodulin-dependent protein kinase II (CaMKII), ERK, and cAMP response element binding protein (CREB) in the hippocampus (Ko et al., 2018). LG promotes neurogenesis within the CNS via Notch-2 signaling pathway inhibition by ERβ dependent learning and memory. LG inhibits expression of Notch-2 mRNA and protein in progenitor cells, which may explain the high number of neurons with overexpression of ERβ (Liu et al., 2010b).

Treatment of ICR mice fed a high-fat diet (HFD) with ILG at 30 and 60 mg/kg/day increased the time spent in the target quadrant where the platform was located in a Morris water maze task. ILG also ameliorated the HFD-induced peripheral insulin resistance, which may results to neuronal damage and cognitive deficits, as measured by decreased plasma insulin and glucose levels. ILG treatment decreased the expression of TNF-α, p-JNK, IL-1β, p-IRSSer307 to reverse inflammation and insulin resistance (Ma et al., 2015). This suggests that ILG has protective activity against the HFD caused learning and memory deficits through inhibition of the TNF-α/JNK/IRS pathway. Based on these findings, LG might be used to enhance cognitive performance in neurological disorders such as AD.

### Anti-Alzheimer’s Activities

Alzheimer’s disease is a chronic NDD and most common cause of dementia characterized by the increasing age-related impairment of learning and memory (Weon et al., 2016). The pathological hallmarks of AD are amyloid beta (Aβ) accumulation, senile plaques, neurofibrillary tangles, dystrophic neurites, and neuronal loss (Lin et al., 2012; Ko et al., 2017). LG in the dose range of 0.2–2 μM to rat hippocampal neurons prevented Aβ_25-35_-induced cell death as shown by 3-(4,5-dimethylthiazol-2-yl)-2,5-diphenyltetrazolium bromide (MTT) and lactate dehydrogenase (LDH) detection assays. LG blocked the significant increase in [Ca^2+^]_i_ and ROS accumulation induced by Aβ_25-35_. LG also exerted an anti-apoptotic role against Aβ_25-35_ toxicity by increasing the Bcl-2 expression LG probably has some neurotropic actions, including increasing the expression of nuclear respiratory factor 3 (Ntf-3) at both the gene and protein levels. LG downregulated Aβ A4 precursor protein-binding family B member 1 (Apbb-1), a peptide that forms the extracellular amyloid fibrils, explaining the mechanism of inhibited Aβ accumulation in rat primary hippocampal neurons (Liu et al., 2009).

Multiple factors can be attributed to the learning and memory deficiency in AD. LG (2.3, 7, and 21 mg/kg/day) effectively attenuated Aβ-induced learning and memory impairment in Wistar rats determined by Morris water maze and shuttle box test. Treatment with LG had no effect on Aβ expression caused by injection of Aβ_25-35_. LG treatment markedly attenuated the CA1 hippocampal neuronal loss in AD model rats as indicated by microtubule-associated protein 2 (MAP2) immunostaining and Nissl’s staining. Moreover, Notch signaling affects CNS, inhibits neuronal differentiation and triggers downstream reactions. The expression of Notch-2 mRNA and protein levels in the AD model rat brains were inhibited by LG treatment (Liu et al., 2010a). Study suggest that LG may serve as a NeuroSERM, an molecule that acts on estrogen mechanisms in the brain while avoiding peripheral estrogen receptors (Liu et al., 2009).

Treatment of Tg2576 mice with LG at 30 mg/kg showed a greater improvement in learning and memory than doses of 3 and 10 mg/kg in Morris water maze and shuttle box test. LG did not alter the levels of amyloid precursor protein (APP); however, levels of an oligomeric form of Aβ protein in Tg2576 mice were significantly reduced in a dose-dependent manner. GFAP levels by immunostaining and immunoblotting were decreased significantly by treatment with 10 and 30 mg/kg LG compared with vehicle treatment in Tg2576 mice. Levels of the active fragment of Notch-2^IC^ were significantly decreased in LQ-treated Tg2576 mice (Liu et al., 2011).

Mitochondria are powerhouses that continuously undergo fusion, fission, transport, and degradation for regulation of their functions. Mitochondrial fission is controlled by dynamin-related protein-1 (DRP-1) while fusion is regulated by mitofuscin 1 and 2 (Mfn1/2) and optic atrophy protein 1 (Opa1). LG treatment of SK-N-MC cells stably expressing YFP resulted in highly elongated and aggregated mitochondria, suggesting that LG induces mitochondrial fusion in a dose- and time-dependent manner (Jo et al., 2016). Pretreatment with LG effectively restored mitochondrial fragmentation in Mfn1 and Mfn2 knockout MEF cells, but had no effect on Opa1 knockout cells. Treatment of SK-N-MC/mito-YFP with LG inhibited Aβ-induced mitochondrial fragmentation. These data suggest that LG treatment might be a best therapeutic strategy against mitochondrial dysfunction (Jo et al., 2016).

Sopolamine is a muscarinic cholinergic receptor antagonist that causes disturbances in the cholinergic system in association with cognitive decline (Haider et al., 2016) by formation of ROS and free radicals (El-Sherbiny et al., 2003). The scopolamine-induced spontaneous alternation behavior, cognitive deficit, and discrimination index of recognition memory were significantly reversed by LG at 20 mg/kg in 4-week-old male CD-1 mice. Scopoamine significantly increased the level of acetylcholinesterase (AChE) and MDA whereas treatment with LG inhibited these increases, suggesting that LG improves cognitive dysfunction. Moreover, LG treatment ameliorated the scopolamine-induced hippocampal expression of BDNF, ERK, and CREB related to cognitive function. Therefore, LG may have potential learning and memory enhancement effects in mice (Ko et al., 2017). The above reports show that the ERβ agonist LG has a promising future as a principal active constituent for the treatment of AD.

### Neuroprotection Against Glutamate-Induced Toxicity

High concentrations of neurotransmitter glutamate can persuade neuronal cell death by receptor-induced cytotoxicity or ROS-mediated oxidative stress, leading to the learning and memory deficits in NDDs (Yang E.J. et al., 2013). Oxidative stress can be induced by 5 mM glutamate in the mouse hippocampal neuronal cell line, HT22, which lacks ionotropic glutamate receptors. Glutamate concentrations greater than millimolar levels can cause oxidative stress through inhibition of cysteine uptake, leading to glutathione reduction in the cells (Murphy et al., 1989).

Pretreatment with LG (10 and 50 μM) and ILG (5 and 10 μM) for 12 h showed protective efficacy against cell cytotoxicity and ROS production induced by 12-h treatment with glutamate. LG effectively recovered the glutamate and calcium chloride (CaCl_2_) toxicity in a concentration-dependent manner (Yang E.J. et al., 2013). The Bid level was increased and phosphorylation of mitogen-activated protein kinases (MAPKs) upon glutamate induction was decreased by 50 μM LG or 5 μM ILG (Yang E.J. et al., 2013; Yang et al., 2016). These results showed that LG and ILG effectively prevented glutamate-induced toxicity by attenuation of mitochondrial dysfunction, and may help to prevent NDDs.

### Neuroprotection Against Stroke

Stroke, with predominant clinical manifestations of ischemia of the brain and hemorrhagic injury, is a major cause of mortality worldwide (Li et al., 2015). The blood-brain barrier (BBB) is a diffusion barrier that selectively blocks influx and efflux of most compounds between blood and brain (Ballabh et al., 2004). However, following a stroke the BBB is disrupted and LG and ILG were detected in plasma and brain tissue with the onset of reperfusion in male Sprague-Dawley rats, suggesting that LG and ILG are able to penetrate the BBB and become distributed in the brain tissue, where they exhibit a protective effect (Li et al., 2015). Zhan and Yang demonstrated that ILG exerted protective effects against rat model of transient middle cerebral artery occlusion (MCAO)-induced focal cerebral ischemia (Zhan and Yang, 2006).

### Neuroprotection Against Brain Glioma

Gliomas are the most common and extremely serious type of primary intracranial neoplasm results in higher mortality and morbidity (Lacroix et al., 2001). The loss of ERβ expression in high-grade glioma tumors suggests the potential tumor suppressor role of ERβ. Treatment of temozolomide (TMZ)-resistant U138 glioma cells with LG significantly increased ERβ expression and synergistically inhibited the U138 glioma cells to TMZ-induced proliferation. In addition, ERβ knockdown or activation of the PI3K/Akt/mTOR pathway by IGF-1 eradicated the function of LG (Liu et al., 2015). These results suggest that the ERβ agonist LG significantly inhibits the PI3K/Akt/mTOR pathways representing a possible therapy for TMZ susceptibility in gliomas.

### Activity Against HIV-1-Associated Neurocognitive Disorders

HIV-1 in CNS results in an array of deficits known as HIV-1-associated neurocognitive disorders (HAND) allied with dendritic simplification and synaptic loss (Antinori et al., 2007). Treatment with HIV-1 transactivating protein (Tat) at 50 nM for 24 h resulted in a significant reduction of F-actin-labeled dendritic puncta and loss of dendrites in brain primary neuronal cultures. LG treatment against Tat 1-86B toxicity protects cell viability, prevents cumulative injury to the dendritic network, and aids recovery from HAND (Bertrand et al., 2014). Results suggest that LG acts via an ERβ-dependent mechanism to avert synaptodendritic damage induced by HIV-1 Tat 1-86B (Bertrand et al., 2014).

### Anti-nociception Activities

Transient receptor potential melastatin 3 (TRPM3) is a calcium-permeable nonselective cation channel member of the large superfamily of TRP ion channels (Straub et al., 2013) expressed in sensory neurons of trigeminal and dorsal root ganglia (DRG). Activation of TRPM3 by the neurosteroid pregnenolone sulfate (PregS) and heat is linked to neuronal myelination (Hoffmann et al., 2010). Incubation of human embryonic kidney 293 (HEK293) cells stably expressing TRPM3 (HEK_mTRPM3_) with LG at 25 μM abrogated PregS (35 μM)-induced [Ca^2+^]_i_ entry. LG elicited a half-maximal block with an IC_50_ of 500 nM. Moreover, LG had no impact on capsaicin (2 μM)-induced transient receptor potential vanilloid-related (TRPV)1 activation, but concentration dependently inhibited allyl isothiocyanate (AITC)-induced TRPA1 activation. In addition, TRPM7-dependent inward or outward currents were affected by LG (20 μM) treatment in HEK293 cells without impairing cell proliferation and viability. Moreover, activation of TRPM3 in DRG neurons is linked to thermosensation. LG at 10 μM strongly counteracted the PregS-induced calcium entry in rat DRG neurons, suggesting that its TRP channel-inhibiting properties and/or TRPM3 inhibition may be useful in the development of novel and tolerable analgesic therapies (Straub et al., 2013).

### Neurogenesis Activities

Recent studies focused on the identification of compounds such as phytoestrogens that are neuro-selective estrogen receptor agonists (NeuroSERMs) (Zhao et al., 2005) and mimic the actions of estrogen in the brain but have insignificant effects on non-neuronal estrogen-responsive tissues (Zhao et al., 2002). ERβ is also reported for learning and memory functions (Liu et al., 2008; Walf et al., 2008). Liu et al. (2010a) reported the effects of LG on newborn Sparague-Dawley rat brain-derived progenitor cells. Study reported that stem and progenitor cells were differentiated into mature neurons by inhibition of Notch proteins (Woo et al., 2009). Decreased Notch-2 mRNA and protein expression in progenitor cells after LG treatment explains the increased neurogenesis and higher number of neurons. In addition, LG promotes neurogenesis by ERβ dependent Notch-2 protein inhibition in SH-SY5Y cells. ERβ gene silencing using RNAi indicated the relationships between Notch receptors, ERs, and neurogenesis (Liu et al., 2010b).

## Other Pharmacological Activities

In addition to the neuropharmacological activities of ILG and LG mentioned above, numerous studies have revealed additional potential therapeutic effects such as radical scavenging, anti-microbial, anti-inflammatory, and antitumor activities.

### Radical Scavenging Activities

Reactive oxygen species induced by oxidative stress result in damage to cellular proteins, lipids, and nucleic acids. RG extract G9315 containing six flavonoids showed good radical scavenging activity against superoxide (O_2_^⋅-^), hydroxyl (^⋅^OH), H_2_O_2_, and singlet oxygen (^1^O_2_) species (Fu and Liu, 1995). In structure-activity relationship studies ILG showed higher activity than LG (Zhang et al., 2012). The ^⋅^OH and ^⋅^OOH radical scavenging activity of ILG was by radical adduct formation and that of LG was through hydrogen atom transfer methods. ILG showed more efficient radical scavenging activity than LG because of its nearly planar conjugated conformation (Wang et al., 2017). LG at 100 μM showed the potential to scavenge 2,2-diphenyl-1-picrylhydrazyl (DPPH) free radicals (Yu et al., 2015).

### Antibacterial Activities

The antibacterial activity of ILG and LG have similar minimal inhibitory concentration (MIC) at 25 μg/ml against *Mycobacterium tuberculosis* (Chokchaisiri et al., 2009). ILG inhibited growth of *Mycobacterium bovis* at 50 μg/ml (Brown et al., 2007), whereas 250 μg/ml was needed for growth inhibition of *Staphylococcus aureus, Staphylococcus epidermidis*, and *Staphylococcus hemolyticus* (de Barros Machado et al., 2005).

### Estrogen Receptor Signaling Activities

The ERα and ERβ are nuclear transcription factors that regulate the transcription of target genes (Paterni et al., 2015). LG in RG shows 20-fold greater affinity for ERβ than ERα determined by competitive binding assays. Although LG binds ERα, it induced minimal transcriptional activation of receptor genes at higher concentration of 2.5 μM. From above results, steroid receptor coactivator-2 (SRC-2) engaged to ER target genes proposing that transcriptional potency and ligand binding are not interrelated after LG treatment (Mersereau et al., 2008). In another study, LG treatment to luciferase reporter plasmid transfected MCF-7 and T47D cells induced a higher differentiation at 5 × 10^-7^ M (Lecomte et al., 2017).

### Anti-inflammatory Activities

Inflammation is an immune system response against harmful stimuli and is characterized by redness, swelling, heat, and pain and macrophages play a vital role in the inflammatory response (Iontcheva et al., 2004). ILG and LG significantly inhibited nitric oxide (NO) production in RAW 264.7 macrophages. LG (30 μM) and ILG (1.6 μM) almost completely suppressed the LPS (1 μg/ml)-induced inducible nitric oxide synthase (iNOS) expression by inhibiting NF-κB/IκBa activation in RAW264.7 macrophages in a dose-dependent manner (Kim J.Y. et al., 2008; Kim Y.W. et al., 2008). In addition, ILG suppressed the LPS-induced phosphorylation of ERK and P38 MAPK, but not JNK1/2 (Kim J.Y. et al., 2008). Treatment of rats with LG (50 mg/kg/day for 3 days p.o. or 15 mg/kg/day for 2 days i.v.) significantly reduced paw swelling induced by carrageenan (Kim Y.W. et al., 2008). These results suggest that LG and ILG have been considered as possible anti-inflammatory agents.

The anti-inflammatory properties of LG were studied in a LPS-stimulated BV-2 microglial cell model and *tert*-butyl hydrogen peroxide (*t*-BHP)-treated ICR male mice model. LG (25, 50, and 100 μM) inhibited LPS-stimulated NO levels in a dose-dependent manner. LG inhibited expressions of iNOS, Cox-2, and pro-inflammatory genes IL-1β and IL-6, but had no effect on TNF-α expression. t-BHP toxicity induced a significant increase in alanine aminotransferase (ALT) and aspartate aminotransferase (AST) levels in serum and TNF-α, IL-1β, IL-6 mRNA expression in the liver of mice, whereas these levels were diminished by LG treatment (Yu et al., 2015). These results suggest that LG inhibits various inflammatory mediators and suppresses inflammation in the liver.

### Anti-periodontitis Activities

Periodontal diseases (gum disease) and dental caries (tooth decay) are the most common human oral infections. *Mutans streptococci* such as *Streptococcus mutans* and *Streptococcus sobrinus* are associated with dental caries through their aciduric, acidogenic, and adhesion properties (Liljemark and Bloomquist, 1996). Different concentrations of ILG and LG were used to determine the MICs for *P. gingivalis, F. nucleatum, P. intermedia, S. mutans*, and *S. sobrinus*. ILG and LG displayed significant growth inhibition in gram-negative periodontal bacteria, but had no effect on gram-positive streptococci. ILG was found to be a effective inhibitor of *P. gingivalis* collagenase and MMP-9 compared with LG. ILG also suppressed dimerization of the inflammatory mediator toll-like receptor 4 (TLR4), which correlated with NF-κB p65 inhibition, activator protein-1 (AP-1) activation and subsequently reduced expression of cytokines in LPS-induced inflammation (Feldman et al., 2011).

### Anti-asthmatic Effects

Asthma is a common long-term inflammatory airway disease characterized by the polarized Th2 cell secretion of Th2 cytokines (Yang E.J. et al., 2013). *G. uralensis* was reported for its protective responsiveness in asthma patients (Wang et al., 1998). An anti-asthma formula, ASHMI^TM^, containing *G. uralensis*, has been reported for improvement in lung function and reduced airway hyperresponsiveness in allergic asthma (Wen et al., 2005). In addition, LG and ILG had high ability in eotaxin secretion suppression compared to glycyrrhizin (Jayaprakasam et al., 2009). Yang N. et al. (2013) reported that the ASHMI formula contains LG, ILG, and 7,4′-DHF. These flavonoids suppress CA-stimulated synthesis of Th2 cytokine and levels of IL-4 and IL-5 in D10 cell culture media supernatants in a concentration-dependent manner without affecting cell viabilities (Yang N. et al., 2013).

### Anti-diabetic Activities

Diabetes is a major metabolic disease with a rapidly increasing prevalence worldwide. In type 2 diabetes, a chronic increase in lipotoxicity and insulin deficiency contributes to increased beta-cell dysfunction and death (Oh, 2015). LG treatment (5 μM) to rat INS-1 insulinoma cells showed increased cell viability and insulin secretion. LG also increased cell viability and decreased apoptosis associated with palmitate (PA)-induced lipotoxicity. In addition, LG suppressed PA-induced endoplasmic reticulum stress markers such as C/EBP homologous protein (CHOP), eukaryotic initiation factor 2α (eIF-2α) and protein kinase RNA-like endoplasmic reticulum kinase (PERK), but had no effect on X-box binding protein (XBP), JNK and activating transcription factor 6 (ATF-6). In addition, LG activated phosphorylation of Akt by ER and subsequently inactivated the PERK pathway, highlighting its therapeutic value in the prevention of diabetes progression (Bae et al., 2018).

Diabetes is characterized by abnormally high blood glucose levels. Oral administration of ILG (10 and 20 mg/kg) to streptozotocin (STZ)-induced diabetic rats for 2 weeks or 2.5 and 5 μM to glucose (30 mM)-insulted N2a cells significantly caused an activation of sirutin (SIRT1, a metabolic sensor of the cellular NAD^+^/NADH ratio) in a dose-dependent manner. In addition, ILG mimics the effects of PGC-1α-mediated mitochondrial biogenesis, calorie restriction, 5′ AMP-activated protein kinase (AMPK)–mediated autophagy and Forkhead box O3a (FOXO3a) mediated stress resistance to counteract experimental diabetic neuropathy (Yerra et al., 2017).

ILG was found to be effective at 100 mg/kg b.w. after 120 min in an oral glucose tolerance test (OGTT), but LG was inefficient in lowering blood glucose levels in the STZ-induced diabetic mice model. In the glucose tolerance test, improvement was observed for ILG at 200 mg/kg b.w. post-administered after 15, 30, 60, 90, and 120 min. Hyperglycemic albino mice treated with 200 mg/kg b.w. of ILG for 14 days showed significant (53%) recovery of random blood glucose level and significant modification of liver glycogen content to 49.92% (Gaur et al., 2014).

### Anti-osteoporosis Activities

Osteoporosis is a disease results from altered bone mass have impact on life expectancy and the quality of life (Lane and Kelman, 2003). Osteoblasts can markedly enhance bone formation (Choi, 2012). LG stimulated cell growth of MC3T3-E1 cells in addition to increasing calcium ion deposition, ALP activity and collagen content, suggesting that LG may induce early and later phases of osteogenic differentiation. The increased osteoblast function was also associated with LG-induced GSH content. In osteoblastic MC3T3-E1 cells, antimycin A (70 μM)–induced production of ROS, cytokines TNF-α and IL-6 and receptor activator of nuclear factor kappa-B ligand (RANKL) was significantly inhibited by treatment with LG (0.04, 0.4, and 4 μM) (Choi, 2012). These data suggest that LG may act as a potential therapeutic candidate against oxidative stress-induced dysfunction in osteoblasts.

### Hepatoprotective Activities

Cadmium (Cd), an environmental heavy metal, largely accumulates in the liver and kidney and induces pulmonary, hepatic, and renal tubular diseases. LG (10–100 μM) treatment to Cd (10 μM) or Cd (1 μM) + buthionine sulfoximine (BSO; 50 μM) exposed H4IIE rat-derived hepatocyte cells showed more active in protecting cells than ILG (0.1–10 μM) treatment. LG also prevented the GSH content reduction induced by BSO, suggesting that LG, but not ILG, have cytoprotection in Cd-induced cellular damages (Kim et al., 2004).

Nitric oxide, an inflammatory mediator, plays a pathophysiological role in various diseases (Colasanti and Suzuki, 2000). Treatment of primary hepatocytes isolated from the liver of male Wistar rats with RG extract and its constituents ILG and LG significantly blocked NO levels in the presence of IL-1β without cytotoxicity. ILG showed an IC_50_ of 11.9 μM. Furthermore, ILG and LG reduced the levels of both iNOS mRNA and protein in primary rat hepatocytes. Moreover, TNF-α and IL-6 mRNA levels were downregulated by ILG and LG, supporting anti-inflammatory effects in hepatocytes (Tanemoto et al., 2015).

The toxicity of acetaminophen, mostly used as analgesic and antipyretic agent, results in massive hepatic necrosis and causes major morbidity and mortality (Zakin and Boyer, 1990). Sprague-Dawley rats were orally administered LG dissolved in 40% PEG administered and then given a single oral dose of acetaminophen in 40% PEG (1.2 g/kg b.w.). LG treatment at 50 mg/kg/day p.o. for 4 days or 15 mg/kg i.v. for 2 days significantly reduced the plasma ALT, improved plasma LDH, and ameliorated liver necrosis induced by acetaminophen. LQ administered i.v. had greater protective effects than p.o. against acetaminophen toxicity (Kim et al., 2006). In addition, LG showed a choleretic effect by increased hepatic bile flow, phase II enzymes and hepatic transporters against in a GalN/LPS-induced rat hepatitis model (Kim et al., 2009).

Phase II drug metabolism enzymes, nuclear receptors and other transcription factors in liver stimulates the toxic substances detoxification and excretion processes. Nuclear factor erythroid 2–related factor 2 (Nrf2) plays major role in drug disposition and phase II drug metabolism enzymes (Zollner et al., 2010). Nrf2 dissociates from Keap1 and translocates into the nucleus leading to expression of target genes under stress conditions. ILG significantly induces Nrf2, UGT1A1, BSEB, MRP2 and GCLS in HepG2 cells. In addition, ILG induces UGT1A1, GCLC, and MRP2 in Nrf2 WT mice but not in Nrf2 KO mice, concluding that ILG has Nrf2-dependent protective activities (Gong et al., 2015).

Hepatocellular carcinoma (HCC) is one of the most common malignant tumors with low recovery rates, and currently available conventional and modified therapies are rarely effective (Kern et al., 2002). Treatment of the human hepatocarcinoma cell line SMMC-7721 with LG resulted in a concentration-dependent decrease in cell viability and apoptosis. The free radical scavenger, *N*-acetyl-cysteine (NAC), suppressed both LG-induced apoptosis and ROS production. Expression of caspase-3, the principal caspase associated with apoptotic nuclear changes, significantly increased in a time-dependent manner, with a 7-fold induction at 72 h after LG (0.4 mM) treatment. Furthermore, LG treatment increased tumor suppressor p53 protein and decreased anti-apoptotic Bcl-2 protein through ROS production and mitochondrial membrane potential loss, in agreement with decreased activities of antioxidant enzymes SOD, GSH, and glutathione peroxidase (GPx) in SMMC-7721 cells. These effects have been proven to be cell-specific as LG at 0.4 mM did not cause significant cell death in human hepatic L-02 cells (Zhang et al., 2009).

### Anti-mutagenic and Anti-cancer Activities

The human osteosarcoma U2OS cell line (which does not express endogenous ERs), human breast MCF-7 cancer cell line (which express ERα), and human cervical HeLa cancer cell line were treated with different doses of LG (0.5, 1, 2, and 2.5 μM). LG treatment at 2 μM stimulated the ERE-tk-luciferase, and ER regulatory elements CECR6, NKG2E, and NKD in ERβ transfected cells due to its greater selectivity, but not with ERα transfected cells. LG (2 mg; 2.5 μl/h) infused for 30 days to nude mice did not involve in uterine enlargement or MCF-7 breast cancer cells induced xenograft (Mersereau et al., 2008). RG extract G9315 containing ILG and LG significantly inhibited the Cytoxan induced mouse bone-marrow micronuclei formation suggesting an antimutagenic activity (Fu and Liu, 1995).

LG was shown to inhibit breast (MDA-MB-231, MCF7, SKBR3) and colon (LoVo, HCT) cancer cells proliferation (Paterni et al., 2015). LG also showed an IC_50_ of 88.3 μM *in vitro* in glioma U87 cells and in an *in vivo* study of U87 glioma cells induced nude mice xenografts (Paterni et al., 2015). ILG treatment blocked the cell growth and induced apoptosis on mouse RCN-9 and human COLO-320DM colon cancer cells (Takahashi et al., 2004). Furthermore, ILG treated at 15 ppm *at libitum* to azoxymethane (AOM; 10 mg/kg b.w. s.c.)-induced colon carcinogenesis in ddY mice inhibited the preneoplastic aberrant crypt foci (ACF) induction (Baba et al., 2002). ILG mixed at 100 ppm in diet fed against AOM (15 mg/kg b.w., 3 times/weekly) induced for 3 weeks effectively reduced the ACF in the colon of male F344 rats (Takahashi et al., 2004).

## Conclusion and Future Prospects

**FIGURE 2:**
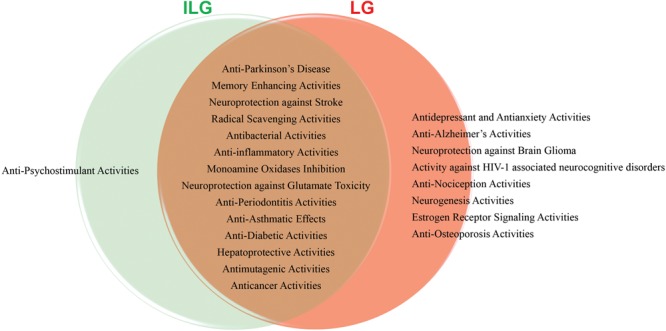
Diagrammatic representation of various pharmacological activities of isoliquiritigenin (ILG) and liquiritigenin (LG).

Aging process is considered major risk factor for age-related diseases such as AD and PD become main concerns in the challenge to improve health. Aging effects cause a cascade of stressors that weaken energy metabolism, mitochondrial functions, and gene expression. The current synthetic drugs treatments to multi-functional age-related NDDs have proven ineffective in curing and have certain side effects. On the other hand, botanical drugs are multicomponent complex systems that might interact with multiple signaling pathways and targets in the human body. This new era on botanical research has provide many opportunities to develop new therapies for patients with age-related diseases. RG have been used widely in traditional medicines throughout the world because of its rich components such as triterpenoids, flavanones, chalcones, coumarines and their glycosides. These active components suggest that RG may be an effective herb for the treatment of different kinds of disease. The main bioactive constituents ILG and LG showed prominent effects in AD, PD and other NDDs models by numerous *in vitro* and *in vivo* experimental studies. The various pharmacological abilities of ILG and LG clearly establish a protective role in human health and disease model(s) (Figure 2). The chemically interconnected structures of ILG and LG attributed for their linked bioactivities offers the greatest potential as therapeutic compounds for age-related degenerative diseases. From this viewpoint, authenticating the traditional herbal remedies will provide clues in the search of new active single specific or combined compounds in the future and scientific evaluation in clinical development use will support their use in health care systems. Therefore, medicinal plants and their active constituents are an excellent source of information in the discovery of new therapeutics to improve human health and lifespan.

## Author Contributions

MR, YL and Y-IL searched the related literature, conceptualized, designed and wrote the manuscript. HK conceptualized, designed the figures in the manuscript. All authors edited and revised the manuscript.

## Conflict of Interest Statement

The authors declare that the research was conducted in the absence of any commercial or financial relationships that could be construed as a potential conflict of interest.
